# Hyperemesis gravidarum and risks of placental dysfunction disorders: a population-based cohort study

**DOI:** 10.1111/1471-0528.12132

**Published:** 2013-01-30

**Authors:** M Bolin, H Åkerud, S Cnattingius, O Stephansson, AK Wikström

**Affiliations:** a Department of Women's and Children's Health, Uppsala UniversityUppsala, Sweden; b Department of Obstetrics and Gynaecology, Sundsvall HospitalSundsvall, Sweden; c Department of Medicine, Solna, Clinical Epidemiology Unit, Karolinska InstituteStockholm, Sweden; d Department of Woman and Child Health, Division of Obstetrics and Gynaecology, Karolinska University Hospital and InstituteStockholm, Sweden

**Keywords:** Hyperemesis gravidarum, placental abruption, pre-eclampsia, small for gestational age, stillbirth

## Abstract

**Objective:**

To study whether pregnancies complicated by hyperemesis gravidarum in the first (<12 weeks) or second (12–21 weeks) trimester are associated with placental dysfunction disorders.

**Design:**

Population-based cohort study.

**Setting:**

Sweden.

**Population:**

All pregnancies in the Swedish Medical Birth Register estimated to have started on 1 January 1997 or later and ended in a single birth on 31 December 2009 or earlier (*n* = 1 156 050).

**Methods:**

Odds ratios with 95% confidence intervals were estimated for placental dysfunction disorders in women with an inpatient diagnosis of hyperemesis gravidarum, using women without inpatient diagnosis of hyperemesis gravidarum as reference. Risks were adjusted for maternal age, parity, body mass index, height, smoking, cohabitation with the infant's father, infant's sex, mother's country of birth, education, presence of hyperthyreosis, pregestational diabetes mellitus, chronic hypertension and year of infant birth.

**Main outcome measures:**

Placental dysfunction disorders, i.e. pre-eclampsia, placental abruption, stillbirth and small for gestational age (SGA).

**Results:**

Women with hyperemesis gravidarum in the first trimester had only a slightly increased risk of pre-eclampsia. Women with hyperemesis gravidarum with first admission in the second trimester had a more than doubled risk of preterm (<37 weeks) pre-eclampsia, a threefold increased risk of placental abruption and a 39% increased risk of an SGA birth (adjusted odds ratios [95% confidence intervals] were: 2.09 [1.38–3.16], 3.07 [1.88–5.00] and 1.39 [1.06–1.83], respectively).

**Conclusions:**

There is an association between hyperemesis gravidarum and placental dysfunction disorders, which is especially strong for women with hyperemesis gravidarum in the second trimester.

## Introduction

Pre-eclampsia, placental abruption, stillbirth and small for gestational age (SGA) birth are associated with abnormal placentation.^1^–^3^ The trophoblast migration into the maternal deciduas and adjacent spiral arteries, which starts in early pregnancy and continues until about the 20th week of gestation, is restricted in these disorders.^4^ Human chorionic gonadotrophin (hCG) is probably an important regulator of this complex process.^5^ There are several variants of hCG. Hyperglycosylated hCG (hCG-H), which is the principal variant of hCG in very early pregnancy, is supposed to be especially important for the stimulation of trophoblast migration.^6^ In the second trimester, hCG-H levels dwindle to less than 1% of total hCG.^6^ Elevated hCG levels in the second trimester could be a result of an insufficient first-trimester trophoblast migration into the spiral arteries, with a subsequent placental hypoxia that stimulates the secretion of other variants of hCG.^5^ Low first-trimester and increased second-trimester hCG levels are associated with later development of pre-eclampsia and SGA.^7^,^8^

Another pregnancy complication associated with high hCG levels is hyperemesis gravidarum,^9^ a severe form of nausea and vomiting. Hyperemesis gravidarum occurs in 0.5–3% of pregnancies and is the most common cause of hospitalisation in the first half of the pregnancy.^10^ In a recent meta-analysis,^11^ an increased risk of SGA was reported in women with hyperemesis gravidarum, but other placental dysfunction disorders were not evaluated. We have found only one study of hyperemesis gravidarum and other placental dysfunction disorders, which supports a weak association between hyperemesis gravidarum and pre-eclampsia risk.^12^

As hCG levels may be both a cause and an effect of placentation, the time of onset of hyperemesis gravidarum may influence the risks of abnormal placentation disorders. In very early pregnancy, high hCG levels may favour normal pregnancy development, whereas, later in pregnancy, abnormal placentation may cause high hCG levels. To our knowledge, no previous study has investigated hyperemesis gravidarum occurring in the first and second trimesters separately, when studying associations with adverse pregnancy outcomes.

In this nationwide population-based study, we estimated the associations between hyperemesis gravidarum and placental dysfunction disorders (pre-eclampsia, placental abruption, stillbirth and SGA). We further studied whether these associations were influenced by the time of hospitalisation for hyperemesis gravidarum.

## Methods

From the Swedish Medical Birth Register (MBR), we included all singleton births born at a gestational age of 22 weeks or more, where the pregnancy was estimated to have started on 1 January 1997 or later and ended in a birth on 31 December 2009 or earlier. The population-based MBR includes prospectively collected information, including demographic data, reproductive history and complications during pregnancy, delivery and the neonatal period, among more than 98% of all births in Sweden.^13^ In Sweden, antenatal care is standardised and free of charge. During the first antenatal visit, usually taking place at the end of the first trimester,^14^ the mother is interviewed about her medical and obstetric history, including height, weight, cigarette consumption and medication. During the study period, one routine ultrasound examination was offered for dating and abnormality screening, commonly in the 17th week of gestation, and approximately 97% of women underwent this examination.^15^ If no early second-trimester ultrasound scan was available, the last menstrual period was used to calculate the gestational age at delivery. Complications during pregnancy and delivery are classified according to the International Classification of Diseases (ICD), as noted by the responsible doctor at discharge from hospital after delivery. Information on each pregnancy and delivery is forwarded to the MBR through copies of standardised antenatal, obstetric and paediatric records. Individual record linkage between the MBR and other registers is possible through each individual's unique personal registration number, assigned to each Swedish resident.^16^

### Study population and exposure variables

The initial study population included 1 156 050 women with singleton births. Our exposure variable was admission to hospital because of hyperemesis gravidarum. Information on exposure was collected through linkage to the nationwide Patient Register, which includes information on dates of hospital admissions and diagnoses, which are classified according to ICD codes. We used the ICD-10 codes O210, O211 and O219 to identify hyperemesis gravidarum. The ICD-10 classification started nationwide in 1997 and the population includes all women who could have been diagnosed with inpatient diagnosis since the introduction of ICD-10. There are no national guidelines for the hospitalisation of women with hyperemesis gravidarum in Sweden, but a general criterion for hospitalisation is the requirement for intravenous fluid. The proportion of women hospitalised as a result of hyperemesis was about the same throughout the study years, which indicates that the handling of these women was constant. We identified 13 287 pregnancies with at least one admission for hyperemesis gravidarum. As severe illness and vomiting later in gestation could be a sign of an adverse outcome rather than hyperemesis gravidarum, pregnancies with a first admission to hospital for the exposure at 22 gestational weeks or later (*n* = 1017) were excluded. The final study population included 1 155 033 pregnant women, 12 270 (1.1%) of whom were exposed to hyperemesis gravidarum before 22 gestational weeks. Pregnancies with hyperemesis gravidarum were further stratified into first-trimester hyperemesis gravidarum, defined as first admission to hospital before 12 completed gestational weeks (*n* = 10 186), and second-trimester hyperemesis gravidarum, with a first admission between 12 and 21 completed gestational weeks (*n* = 2084). When analysing the risk of SGA, we excluded stillbirths and births with missing or misclassified information on birthweight and/or gestational age (*n* = 5036), resulting in 1 146 142 births.

### Outcome variables

The outcomes studied were pre-eclampsia, placental abruption, stillbirth and SGA birth. Pre-eclampsia was identified by the ICD-10 codes O14–15. We had no information on gestational age at onset of pre-eclampsia in the MBR. We therefore categorised pre-eclampsia by time of birth into preterm (birth before 37 gestational weeks) and term (birth at 37 gestational weeks or later) pre-eclampsia. Pre-eclampsia was defined as a blood pressure of more than or equal to 140/90 mmHg, combined with proteinuria (≥0.3 g/24 hours) occurring after 20 weeks of gestation. The quality of the diagnosis of pre-eclampsia in MBR has been validated previously: of 148 pregnancies coded as pre-eclampsia in MBR, 137 (93%) had the disease according to the individual records.^17^ Placental abruption was identified by ICD-10 code O45. The clinical definition of placental abruption is premature separation of the placenta. Stillbirth was defined as a fetal death occurring at 28 weeks of gestation or later. SGA was defined as a birthweight of two standard deviations or more below the mean birthweight for gestational age according to the sex-specific Swedish fetal growth curve.^18^ We defined births with a birthweight for gestational age of five or more standard deviations above or below the mean for a given gestational age as misclassified.

### Covariates

Information on maternal body mass index (BMI), height, cigarette consumption and cohabitation with the infant's father was collected from the first antenatal visit. Data on maternal age, parity and the infant's sex were collected at delivery. To obtain information on the mother's country of birth and the highest level of formal education, individual linkage with the Register of Total Population and the Education Register (31 December 2010) was performed. Information on the presence of maternal hyperthyreosis was collected at discharge from the delivery hospital using the ICD-10 code E05. Further, information on pregestational diabetes and chronic hypertension was collected from the first antenatal visit and at discharge from the delivery hospital (ICD-10 codes E10–14 and O241–243 for diabetes and O10–11 for hypertension). The covariates were categorised according to Table 1.

**Table 1:** Sociodemographic and clinical characteristics of women giving birth to a singleton infant in Sweden between 1997 and 2009 by hospitalisation for hyperemesis gravidarum

Maternal characteristic	Number	Hyperemesis		
		No	Yes	
		(*n* = 1 142 763)	First trimester (*n* = 10 186)	Second trimester (*n* = 2084)
		Rate (%)	Rate (%)	Rate (%)
**Age (years)**				
<25.0	172 336	14.9	22.1	29.1
25.0–29.9	358 454	31.1	34.2	33.9
30.0–34.9	400 752	34.8	28.8	23.9
>35.0	221 216	19.2	15.0	13.1
Data missing	2275			
**Parity**				
1	512 358	44.4	42.2	51.5
2–3	573 721	49.7	49.8	42.7
≥4	68 954	6.0	8.1	5.8
**BMI (kg/m^2^)**				
<18.5	23 728	2.3	6.6	5.1
18.5–24.9	621 409	61.5	62.4	55.5
25.0–29.9	254 741	25.2	21.7	25.2
>30.0	110 802	11.0	9.3	14.2
Data missing	144 353			
**Height (cm)**				
100–161	236 543	21.9	30.2	26.2
162–171	609 875	56.8	52.8	55.0
≥172	228 443	21.3	17.0	18.8
Data missing	80 172			
**Smoking (cigarettes/day)**				
0	982 936	90.5	96.9	92.8
1–9	73 935	6.8	2.4	5.7
≥10	28 547	2.6	0.7	1.4
Data missing	69 615			
**Living with infant's father**				
Yes	1 029 104	97.7	96.2	96.2
No	24 249	2.3	3.8	3.8
Data missing	101 631			
**Infant's sex**				
Female	560 285	48.4	55.5	50.3
Male	594 489	51.6	44.5	49.7
Data missing	258			
**Mother's country of birth**				
Nordic	943 877	83.0	59.2	69.6
Non-Nordic	197 131	17.0	40.8	30.4
Data missing	14 025			
**Education (years)**				
≤9	108 784	9.7	18.5	18.0
10–12	478 942	42.9	46.3	47.0
13–14	159 319	14.3	13.2	11.8
≥15	367 775	33.1	22.0	23.3
Data missing	40 213			
**Hyperthyreosis**				
Yes	565	0.05	0.18	0.10
No	1 154 468	99.95	99.82	99.90
**Pregestational diabetes**				
Yes	7353	0.6	0.8	1.3
No	1 147 680	99.4	99.2	98.7
**Chronic hypertension**				
Yes	6971	0.6	0.4	0.8
No	1 148 062	99.4	99.6	99.2

### Statistical analysis

The risks of pre-eclampsia, placental abruption, stillbirth and SGA were calculated for women admitted to hospital for hyperemesis gravidarum, using women without admission for hyperemesis gravidarum as reference. Odds ratios (ORs) with 95% confidence intervals (CIs) were calculated using the generalised estimation equation (PROC GENMOD) method, as observations are not independent in women who delivered more than once during the study period. Adjustments were made for maternal factors associated with risks of the exposure and outcomes, including maternal age, parity, BMI, height, smoking, cohabitation with the infant's father, infant's sex, mother's country of birth, education, presence of hyperthyreosis, pregestational diabetes and chronic hypertension. Further, we adjusted for the year of infant birth, categorised into 1997–2001, 2002–2005 and 2006–2009.

All statistical analyses were performed with Statistical Analysis Software version 9.1 (SAS Institute, Inc., Cary, NC, USA).

## Results

In pregnancies with hyperemesis gravidarum, mothers with a first admission to hospital in the first trimester were generally older, more often multigravida, of normal weight (BMI < 25.0), nonsmokers, expecting a female infant and from a non-Nordic country than were women with a first admission to hospital in the second trimester (Table 1).

Compared with pregnancies without hyperemesis gravidarum, pregnancies with hyperemesis gravidarum had a slightly increased risk of pre-eclampsia, especially preterm pre-eclampsia. When stratifying the exposure into first- and second-trimester hyperemesis gravidarum, the strongest association between hyperemesis gravidarum and pre-eclampsia was observed between second-trimester hyperemesis gravidarum and preterm pre-eclampsia, where a more than twofold increased risk was seen (Table 2).

**Table 2:** Hyperemesis gravidarum and risk of pre-eclampsia

Hyperemesis gravidarum	Pre-eclampsia								
	Preterm or term			Preterm (<37 weeks)			Term (≥37 weeks)		
	No.	Rate (%)	AOR* (95% CI)	No.	Rate (%)	AOR* (95% CI)	No.	Rate (%)	AOR* (95% CI)
No	31 847	2.8	Reference	7322	0.6	Reference	24 525	2.1	Reference
Yes	374	3.0	1.19 (1.05–1.34)	101	0.8	1.36 (1.09–1.70)	273	2.2	1.13 (0.98–1.30)
First trimester (<12 weeks)	294	2.9	1.17 (1.02–1.34)	72	0.7	1.19 (0.91–1.55)	222	2.2	1.16 (0.99–1.35)
Second trimester (12–21 weeks)	80	3.8	1.26 (0.98–1.63)	29	1.4	2.09 (1.38–3.16)	51	2.4	1.01 (0.73–1.40)

AOR, adjusted odds ratio; CI, confidence interval.

* Adjustments were made for maternal age, parity, body mass index, height, smoking, cohabitation with infant's father, infant's sex, mother's country of birth and years of formal education, presence of hyperthyreosis, pregestational diabetes or chronic hypertension, and year of birth of infant.

Compared with pregnancies without hyperemesis gravidarum, pregnancies with hyperemesis gravidarum were associated with an almost 50% increased risk of placental abruption and a slightly increased risk of an SGA birth. When stratifying the exposure into first- and second-trimester hyperemesis gravidarum, the strongest risks were again observed for second-trimester hyperemesis gravidarum, which was associated with a more than threefold increased risk of placental abruption and a 39% increased risk of SGA. First-trimester hyperemesis gravidarum was not significantly associated with placental abruption and SGA. We did not find any associations between hyperemesis gravidarum and stillbirth risks (Table 3).

**Table 3:** Hyperemesis gravidarum and risk of placental abruption, stillbirth and giving birth to a small for gestational age (SGA) infant

Hyperemesis gravidarum	Placental abruption			Stillbirth*			Small for gestational age**		
	No.	Rate (%)	AOR*** (95% CI)	No.	Rate (%)	AOR*** (95% CI)	No.	Rate (%)	AOR*** (95% CI)
No	4652	0.4	Reference	3628	0.3	Reference	26 683	2.4	Reference
Yes	64	0.5	1.47 (1.10–1.95)	41	0.3	0.99 (0.68–1.44)	381	3.1	1.18 (1.04–1.33)
First trimester (<12 weeks)	42	0.4	1.13 (0.80–1.61)	35	0.3	0.95 (0.62–1.44)	298	2.9	1.13 (0.99–1.30)
Second trimester (12–21 weeks)	22	1.1	3.07 (1.88–5.00)	6	0.3	1.18 (0.53–2.64)	83	4.0	1.39 (1.06–1.83)

AOR, adjusted odds ratio; CI, confidence interval.

* Only births ≥28 gestational weeks were included.

** Defined as a live-born infant with a birthweight for gestational age of less than two standard deviations below the sex-specific Swedish fetal growth curve.^18^

*** Adjustments were made for maternal age, parity, body mass index, height, smoking, cohabitation with infant's father, infant's sex, mother's country of birth and years of formal education, presence of hyperthyreosis, pregestational diabetes or chronic hypertension, and year of birth of infant.

## Discussion

### Main findings

Our nationwide study showed an increased risk of pre-eclampsia, placental abruption and SGA birth in women presenting with hyperemesis gravidarum. Risks were particularly associated with hospitalisation for hyperemesis gravidarum in the second trimester. As pre-eclampsia, placental abruption and SGA birth are associated with abnormal placentation,^1^,^2^ our findings indicate an association between abnormal placentation and hyperemesis gravidarum manifested in the second trimester.

### Strengths

The major strengths of this study include the nationwide population-based design, where data were collected prospectively, precluding recall bias. The large sample size made it possible to stratify hyperemesis gravidarum by trimester of hospitalisation, and to study rare adverse outcomes, such as preterm pre-eclampsia and placental abruption. Control for a substantial number of possible confounders, such as BMI, socioeconomic status, smoking and certain maternal chronic diseases, was made. Such maternal characteristics have previously largely explained the increased risks of poor pregnancy outcomes, at least SGA births, in pregnancies with hyperemesis gravidarum.^10^ However, it cannot be excluded that the findings may partly be a result of unmeasured confounding.

### Weaknesses

One weakness of the study is the lack of a consensus on the definition of severe hyperemesisis, and the fact that we did not have information on the start of hyperemesis gravidarum symptoms, apart from the information on the date of first admission to hospital. We did not have information on maternal diet, weight loss or insufficient weight gain during pregnancy. Weight loss/low weight gain during pregnancy have been shown to be risk factors for delivery of an SGA infant, but have a negative association with pre-eclampsia risk.^19^ In contrast with the pre-eclampsia risk, the finding that hyperemesis gravidarum increases the risk of SGA might partly be mediated by insufficient weight gain in pregnancies with hyperemesis gravidarum.

### Interpretation

The finding of an increased risk of pre-eclampsia in women with hyperemesis gravidarum is in agreement with one former study.^12^ However, the present study has increased the knowledge on the association between the disorders, as we separated preterm from term pre-eclampsia. Pre-eclampsia with an early onset has been proposed to have a stronger association than late-onset pre-eclampsia with inadequate and incomplete spiral artery remodelling.^20^,^21^ The finding of a stronger association between hyperemesis gravidarum in the second trimester and preterm pre-eclampsia suggests that hyperemesis gravidarum could be associated with abnormal placentation.

Several earlier studies have shown an association between hyperemesis gravidarum and SGA birth.^10^,^22^ A recent meta-analysis found a 28% increased risk of SGA birth in women with hyperemesis gravidarum, albeit with significant heterogeneity across the included studies.^11^ Our findings are in agreement with this conclusion, and the results add that hyperemesis gravidarum in the second trimester seems to be slightly more strongly associated with SGA birth than is hyperemesis gravidarum in the first trimester.

We did not find any former study of the risks of placental abruption in pregnancies with a diagnosis of hyperemesis gravidarum. An association between placental abruption and hyperemesis gravidarum was found in this study, but the increased risk was only seen in pregnancies hospitalised for hyperemesis in the second trimester. In this population, there was no association between stillbirth and hyperemesis gravidarum. This could be because stillbirth is a very rare condition and the sample size was too small to show an association.

Earlier studies have reported an association between high levels of total hCG or β-hCG in the second trimester and the risk of pre-eclampsia and SGA birth.^8^,^23^ Our findings are in agreement with these studies, as hyperemesis gravidarum is associated with high levels of hCG.^9^ The high levels of hCG in the second trimester could be a compensatory mechanism for an insufficient early trophoblast migration and invasion of the spiral arteries.^5^ Thus, abnormal placentation may cause high levels of hCG in the second trimester, which then cause hyperemesis gravidarum with a late onset.

However, there are other possible explanations for the results. Hyperemesis gravidarum is a classical example of an interaction of biological and psychosocial factors.^24^ There are associations with both high thyroxin and estradiol levels.^25^ hCG is structurally similar to thyroid-stimulating hormone and an increased production of thyroxin is associated with hyperemesis gravidarum. Women with second-trimester hyperemesis might have a prolonged or delayed stimulation of thyroxin, compared with women with first-trimester hyperemesis. This might affect placentation, as former studies have shown an association between hyperthyreosis and placental dysfunction disorders.^26^ Pre-eclampsia is mostly associated with low estradiol levels,^27^ and we therefore doubt that estradiol is a link between these disorders.

## Conclusions

In conclusion, this study has demonstrated associations between hyperemesis gravidarum diagnosed in the second trimester and placental dysfunction disorders, i.e. preterm pre-eclampsia, placental abruption and SGA. The clinical take-home message of our findings is that pregnancies with hyperemesis gravidarum in the second trimester demand an increased alertness and supervision for the development of adverse outcomes associated with abnormal placentation. Further study is required to determine whether prophylactic treatment with low-dose aspirin, controls of uterine artery Doppler and increased surveillance of blood pressure and fetal growth should be considered in these women.
